# Editorial: Pattern recognition receptors at the crosstalk between innate and adaptive immune systems and implications for vaccine development

**DOI:** 10.3389/fcimb.2022.1088029

**Published:** 2022-12-02

**Authors:** Omar Rossi, Pietro Mastroeni

**Affiliations:** ^1^ GSK Vaccines Institute for Global Health, Siena, Italy; ^2^ University of Cambridge, Cambridge, United Kingdom

**Keywords:** vaccine, pattern recognition receptors (PPR), innate immunity, adjuvants, adaptive immunity, PAMP

The increase in prevalence of multi-drug resistant bacterial pathogens, and the emergence of new viruses that can cause damaging pandemics, highlight the urgent need for effective, easy-to-develop and affordable vaccines.

The cross talk between innate and acquired immunity is pivotal for the development of balanced immune responses with the appropriate qualitative and quantitative traits.

The type of vaccine (e.g., live attenuated, killed whole cell, subunit, nucleic acid-based), its chemical composition, and the adjuvant molecules/structures used to enhance or modulate the immune response, determine the dynamic engagement with immune cells and receptors in a complex and multifaceted process. This results in the initiation of a crosstalk between innate and acquired immunity, hopefully leading to what we know as vaccine-mediated protective immunity against infection.

Pattern Recognition Receptors (PRRs) are key molecules in triggering immunity and inflammation. These are transmembrane or cytosolic glycoproteins expressed in many cell types including macrophages and dendritic cells. One of their primary roles is to detect any loss of homeostasis in the body and “raise the alarm”. They do this by recognizing exogenous motifs that are highly conserved and frequently associated with pathogens, known as Pathogen-Associated Molecular Patterns (PAMPs), or endogenous molecules released upon tissue damage, known as Danger-Associated Molecular Patterns (DAMPs). Upon activation, PRRs initiate a cascade of signaling events that culminate into microbicidal and pro-inflammatory responses to eliminate, or at least to contain, the threat. Part of the pro-inflammatory response elicited by PRR activation in innate immune cells is the production of cytokines, such as TNF-α, IL-6, IL-12, IL-1β and IL-18.

The ability of PRRs to enhance immune responses has led to the exploration of PRR ligands as adjuvants in vaccine preparations. This is the case of monophosporyl lipid A [contained in AS01 (Didierlaurent et al., 2017)] which activates Toll-like Receptor (TLR) 4, or the TLR7 agonist AS37 (Gonzalez-Lopez et al., 2019), lipoproteins recognized by TLR2, and bacterial flagellin by TLR5. Indeed, PRR engagement determines changes in antigen presenting cells to make them more efficient at the stimulation of naïve T cells; this process leads to their differentiation into the most appropriate subset of activated T cells, that will in turn guide the immune system towards the containment and eventual elimination of the pathogen.

However, there is a fine balance between achieving beneficial adjuvanticity and causing catastrophic reactogenicity; for example, excessive activation of TLR4 can cause endotoxic shock and sepsis; mRNA is known to activate TLR3, TLR7 and TLR8 inducing severe inflammatory responses.

The possibility to reduce detrimental engagement with PRR has been recently exploited in the generation of modern vaccines. For example, mRNA-based COVID-19 vaccines are usually modified to contain N1-methyl-pseudouridine in order to achieve reduced activation of TLRs (e.g. TLR3) and therefore reduced reactogenicity (Nance and Meier, 2021).

Similarly, self-adjuvanting vaccines with ability to present antigens in a natural conformation at high yield, known as Generalized Modules for Membrane Antigens (GMMA) (Berlanda Scorza et al., 2012) or Outer Membrane Vesicles (OMV) can be obtained from Gram-negative bacteria. GMMA and OMV are exosomes derived from the bacterial outer membrane. These are easily purified from bacteria genetically modified to increase the blebbing and to reduce the risk of inducing systemic reactogenicity in humans. Reduction in reactogenicity is commonly achieved through modification of the lipid A portion of lipopolysaccharide (detoxification).

For example, *Salmonella* GMMA have been produced from bacteria harboring deletions in genes encoding enzymes responsible for the acylation of LPS (*msbB*, *pagP*); this leads to the less reactogenic penta-acylated LPS being contained within the vesicles rather than the wild-type hepta-acylated LPS (Rossi et al., 2016). These vaccines have been proven to be immunogenic and efficacious in animal models (Micoli et al., 2018; Gasperini et al., 2021). Similar approaches are being used for the development of novel vaccines against *Shigella*, where GMMA from bacteria with *msbB or htrB* deletions (Rossi et al., 2014; Gerke et al., 2015) were safe (Launay et al., 2017; Obiero et al., 2017) and able to elicit functional antibodies in humans (Micoli et al., 2021; Kapulu et al., 2022). Other examples of bacterial vaccines based on this strategy are those against *Neisseria meningitidis*, with pentacylated LPS obtained *via* deletion of *lpxL1*, which have been tested preclinically (Koeberling et al., 2014) and clinically (Keiser et al., 2011). The licensed and widely used Bexsero vaccine against group B-meningococci also relies on adjuvating properties of OMV, where detoxification of lipid A is achieved chemically rather than genetically (Rappuoli et al., 2018).

Genetically or chemically detoxified vaccines still retain some ability to engage PRRs.

Indeed, GMMA from *Shigella sonnei* with detoxified lipid A still induce secretion of cytokines/chemokines in cultured human peripheral blood mononuclear cells and can activate expression of markers specific for monocytes, dendritic, Natural Killer, B, and γδ T cells (Tondi et al.). This indicates that some residual ability to engage PRR is still compatible with reduced reactogenicity and it still occurs predominantly *via* TLR4 engagement (Piccioli et al., 2022).

The examples above indicate that a partial reduction in reactogenicity is sufficient to maintain the balance between tolerability and retention of immunogenicity. Further research will be crucial to determine to which extent it is possible to reduce interaction of vaccines with PRR without impairing their immunogenicity.

Stimulation of innate immunity is necessary also for the efficacy of gyco-conjugates; here a better understanding of the mechanism of carbohydrate interaction with innate and adaptive immune cells will benefit the design of a new generation of glycan-based vaccines and of immunomodulators to fight both longstanding and emerging diseases (Stefanetti et al.).

Despite decades of research in the relationships between PRRs activation, vaccine immunogenicity and safety, there is still enormous room for improvement in this aspect of vaccinology. Progress of this area will need to stem from a close collaboration between fundamental vaccine scientists, those involved in development and manufacturing and scientists focused on the fundamental mechanisms of PRRs-mediated immune responses. Efficacy and safety are the essential pre-requisites for development of vaccines and adjuvants. Therefore, the balance between reductions in engagement of PRRs and retention of immunogenicity must be carefully designed.

However, logistics concerning manufacturing, scale up, ease of production and cost will certainly determine whether new fundamental approaches have a real potential to stem into practical applications. Ideal vaccines would be those where the residual intrinsic ability to engage PRR would not cause significant reactogenicity, but would be sufficient to trigger immune responses, in the absence of additional adjuvants.

## Author contributions

Both authors contributed to writing and conceiving the manuscript. All authors contributed to the article and approved the submitted version.
